# P-1017. Key Fungal Secreted Proteases in Coccidioidomycosis

**DOI:** 10.1093/ofid/ofae631.1207

**Published:** 2025-01-29

**Authors:** Christina Homer, Elena Ochoa, Mark Voorhies, Anita Sil

**Affiliations:** UC San Francisco, San Francisco, California; UC San Francisco, San Francisco, California; UC San Francisco, San Francisco, California; UC San Francisco, San Francisco, California

## Abstract

**Background:**

*Coccidioides*, a dimorphic fungus endemic to the Southwest United States, causes unacceptably high morbidity/mortality. *Coccidioides’* virulence stems from its unique and poorly understood host form, the spherule. Spores are inhaled by the host and develop into spherules, which mature until they fill with hundreds of endospores and then rupture, disseminating endospores throughout the host. This entire process is known as spherulation. As spherules are unique to *Coccidioides*, most spherule biology remains unknown. *Coccidioides* genomes encode 2 expanded families of proteases, subtilases and deuterolysins, that we hypothesize are involved in spherulation and are promising treatment targets.

**Methods:**

We took 3 approaches to characterize proteases in spherule biology. We studied spherulation by microscopy in the presence of AEBSF (serine protease inhibitor) and 1,10-phenanthroline (metalloprotease inhibitor). We profiled the *Coccidioides* transcriptome during spherulation, in Converse media, at 39˚C, 10% CO_2_, or during hyphal growth, in Converse media at room temperature. Using multiplex substrate profiling-mass spectrometry on supernatants from protease deletion mutants or wildtype, we characterized the total amount of secreted protease activity and amino acid preferences around sites of proteolysis.

**Results:**

Treatment of *Coccidioides* with a protease inhibitor that blocks subtilase activity prevented spherule formation, indicating a role for subtilases in spherulation. Subtilase and deuterolysin expression is induced upon spherule formation in 4 patterns: 1) during early spherule formation, 2) continuous increase over spherulation, 3) 2 peaks of expression in arthroconidia and endospores, or 4) dramatic increase during endospore release. The subtilase Sub1 fits the 4th pattern and is a top 10 most abundant transcript in spherules during endospore release. Sub1 is a major contributor to secreted protease activity in spherules and exhibits a preference for hydrophobic substrates. Phenotypic characterization of this mutant is underway, including virulence studies.

**Conclusion:**

By linking protease activity to spherulation, we have demonstrated the importance of further dissecting the role of these 2 expanded protease families in *Coccidioides* virulence.

**Disclosures:**

**All Authors**: No reported disclosures

